# Systematic Transcriptome Analysis of Noise-Induced Hearing Loss Pathogenesis Suggests Inflammatory Activities and Multiple Susceptible Molecules and Pathways

**DOI:** 10.3389/fgene.2020.00968

**Published:** 2020-08-28

**Authors:** Quan Wang, Yilin Shen, Haixia Hu, Cui Fan, Andi Zhang, Rui Ding, Bin Ye, Mingliang Xiang

**Affiliations:** ^1^Department of Otolaryngology & Head and Neck Surgery, Ruijin Hospital, Shanghai Jiao Tong University School of Medicine, Shanghai, China; ^2^Ear Institute, Shanghai Jiao Tong University School of Medicine, Shanghai, China

**Keywords:** systematically evaluation, diagnostic value, NIHL pathogenesis, spiral ganglion neurons, functional annotation

## Abstract

Noise-induced hearing loss (NIHL) is characterized by damage to cochlear neurons and associated hair cells; however, a systematic evaluation of NIHL pathogenesis is still lacking. Here, we systematically evaluated differentially expressed genes of 22 cochlear samples in an NIHL mouse model. We performed Kyoto Encyclopedia of Genes and Genomes (KEGG) pathway enrichment analysis and weighted gene co-expression network analysis (WGCNA). Core modules were detected using protein–protein interactions and WGCNA with functional annotation, diagnostic value evaluation, and experimental validation. Pooled functional annotation suggested the involvement of multiple inflammatory pathways, including the TNF signaling pathway, IL-17 signaling pathway, NF-kappa B signaling pathway, rheumatoid arthritis, and p53 signaling pathway. The core modules suggested that responses to cytokines, heat, cAMP, ATP, mechanical stimuli, and immune responses were important in NIHL pathogenesis. These activities primarily occurred on the external side of the plasma membrane, the extracellular region, and the nucleus. Binding activities, including CCR2 receptor binding, protein binding, and transcription factor binding, may be important. Additionally, the hub molecules with diagnostic value included Relb, Hspa1b, Ccl2, Ptgs2, Ldlr, Plat, and Ccl17. An evaluation of Relb and Hspa1b protein levels showed that Relb was upregulated in spiral ganglion neurons, which might have diagnostic value. In conclusion, this study indicates that the inflammatory response is involved in auditory organ changes in NIHL pathogenesis; moreover, several molecules and activities have essential and subtle influences that have translational potential for pharmacological intervention.

## Introduction

Noise-induced hearing loss (NIHL) threatens workers in many occupations, and can also affect those exposed to loud music and other recreational activities, such as concerts, clubs, and bars (Clark and Bohne, 1999; Basner et al., 2014). In 2015, the World Health Organization estimated that more than a billion teenagers and young adults suffer from NIHL, which is caused by excess exposure to loud sounds without adequate hearing protection (World Health Organization, 2015).

The biology and epidemiology of NIHL are still under extensive investigation, and data from animal models have shown that several distinct structures within the cochlea are involved (Lynch and Kil, 2005). Intense noise exposure can result in reversible or irreversible hearing loss, including temporary threshold shift (TTS) or permanent threshold shift (PTS). Both TTS and PTS involve damage to or loss of multiple cellular structures within the cochlea, including hair cells, supporting cells, spiral ganglion neurons, and cells within the stria vascularis and spiral ligament. Functional and structural damage is thought to comprise a cascade triggered by reactive oxygen/nitrogen species (ROS/RNS) and anti-ROS/RNS (Yamane et al., 1995; Yamasoba et al., 1998). Glutathione peroxidase 1 (GPx1) is an enzyme that catalyzes glutathione (GSH) to balance ROS levels, and may be essential to maintain normal cochlear function. Glutathione peroxidase 1 is highly expressed in the cochlea of mammals (Kil et al., 2007) and is deleted after noise exposure (Ohlemiller et al., 1999). In support of this hypothesis, mice with GPx1 deletion have shown increased NIHL vulnerability relative to their wild-type littermates (Ohlemiller et al., 2000). Notably, the safety and efficacy of a novel GPx1 mimic was observed in a randomized, double-blind, placebo-controlled phase 2 clinical trial for the prevention of NIHL (Kil et al., 2017). Similarly, the proliferation of peroxisomes and auditory dysfunction was rescued when the gene was restored in pejvakin-deficient mice (Delmaghani et al., 2015).

Although NIHL is characterized by damage to cochlear neurons and associated hair cells (Kurabi et al., 2017), we have yet to obtain a systematic understanding of the molecules and pathways involved. Here, we aimed to analyze differences in the gene profiles of cochlea from wild-type and noise-exposed mice, to detect changes in important molecules and pathways. An understanding of the specific molecules and pathways would significantly facilitate the advancement of future studies, health care, and clinical work.

## Materials and Methods

### Data Preparation, Extraction, and Processing

Two authors performed an independent study retrieval from the Gene Expression Omnibus (GEO)^1^ and ArrayExpress (AE)^2^ databases for NIHL mRNA expression profiling studies. The medical subject terms included (”noise” [MeSH Terms] OR noise [All Fields]) AND (”hearing loss” [MeSH Terms] OR hearing loss [All Fields]). We limited the series study type and a 5+ sample size to increase credibility, and the two databases were screened for title, summary, general design, and description. We systematically compared the values of cochlear mRNA between noise-exposed mice and healthy controls following full-text dataset examinations and consensus meetings. Our results were validated prior to formal data extraction and processing according to previous studies (Zhang et al., 2018). Quantile normalization, log2 transformation, official gene symbol translation, and grouping annotation were saved as series matrix files for qualified studies.

After the batch effect was removed, susceptible mRNAs or differentially expressed genes (DEGs) were determined by the area under curve (AUC) and p value criteria. The *p*-value cutoff was set to 0.05, and the best threshold was calculated using Youden’s J statistic (Youden, 1950; Robin et al., 2011). The maximum distance to the diagonal line was considered as the optimal cutoff point value.

### Establishment of Gene Co-expression Network

As an efficient system biology method, weighted gene co-expression network analysis (WGCNA) can construct a scale-free network using gene expression data (Horvath and Dong, 2008). Experiments were conducted in accordance with the criteria of outlier samples detection and appropriate soft threshold power selection. Module identification procedures included topological overlap matrix (TOM) formation representing adjacency, hierarchical gene clustering with a deep-split value of 2, a minimum size cutoff of 30, and similar modules merging with a height cutoff of 0.4. All procedures above were computed with the module preservation function implemented in the WGCNA package (Langfelder and Horvath, 2008; Miao et al., 2018).

### Module Detection and Functional Annotation

Weighted gene co-expression network analysis module detection was performed following conventional procedures. Briefly, candidates were modules with high membership scores (module-trait correlation) and genes with high significance scores (gene-trait correlation). The gene significance cutoff was set at 0.2, and the module membership score at 0.5, with a threshold *p*-value <0.05 (Horvath and Dong, 2008).

Protein-protein interaction (PPI) modules were performed in accordance with a previous study (Wang et al., 2018). Briefly, DEGs were processed for identification of the most significant interactive associations (a combined score >0.4) using the Search Tool for the Retrieval of Interacting Genes (STRING)^3^ and significant cluster calculation using the Cytoscape (version 3.6.1) software (Shannon et al., 2003). Molecular Complex Detection application. Cutoff of false degree, node score, haircut, false K-score, and max depth from seed were set to 2, 0.2, true, 2, and 100, respectively.

### Noise Exposure

Five-week-old mice were exposed to 10 kHz octave band noise at 108 dB sound pressure for 2 h, as previously described (Kujawa and Liberman, 2009). Briefly, mice were placed in a circular cage with four fan-shaped compartments and could move within the compartment. Setup included a soundproof chamber for cage placement, soundproofing acoustical foam for reflection minimization, a Fostex FT17H Tweeter Speaker for noise recordings at the top of the sound chamber, and a B&K sound level meter for calibration of a variation of 1.5 dB across the cage.

### Histopathological Analysis

After temporal bone division, the cochlea was excised, immersed in a fixative containing 4% paraformaldehyde in phosphate buffered saline solution for 1 day, and decalcified in 10% EDTA for 7 days. Specimens were sliced into 3 μm sections and mounted onto silane-coated slides, stained, and observed under a light microscope. The evaluation of cochlear histology included three regions (apical, middle, and basal) in the Rosenthal canal. Our sample size included five per group, and every fifth modiolar section (a total of five) was subjected to histopathological assessment. The same animals were used for immunohistochemistry (IHC) staining.

Primary antibodies against the following antigens were applied: Relb (Abcam, ab180127), Hspa1b (Abcam, ab5442), and Caspase 3 (Abcam, ab44976).

### Statistical Analysis

Computations and data visualization were performed using Python 3.7 or R 3.5.1. R packages included clusterProfiler (version 3.8.1), GGally (version 1.4.0), limma (version 3.36.5), network (version 1.13.0.1), pheatmap (version 1.0.10), pROC (version 1.12.1), Rtsne (version 0.13), rms (version 5.1-2), sna (version 2.4), and WGCNA (version 1.66). Significance was defined as *p* < 0.05.

## Results

### Systematic Evaluation of DEGs in NIHL

We retrieved suitable studies, which included nine relevant mRNA expression datasets in GEO and one in AE. After duplicates were removed, the title description, summary information, and overall design of the remaining nine studies (E-GEOD-8342, GSE100365, GSE81667, GSE85290, GSE72722, GSE59416, GSE59415, GSE65249, and GSE12810) were reviewed. According to the inclusion principle, three studies (EGEOD8342, GSE100365, and GSE12810) were enrolled for the subsequent assessment. In total, 22 samples (clinical traits indicated in Supplementary Table S1A) were included in our study. Among them, 11 (50%) were from experimental groups, and 11 (50%) were from normal controls. The number of mRNAs among the included platforms ranged from 9,131 to 21,602 (Supplementary Figure S1A). Assessment of these samples was conducted using the tSNE algorithm. Among the 22 samples, notable outliers were not detected (Supplementary Figure S1B).

**FIGURE 1:**
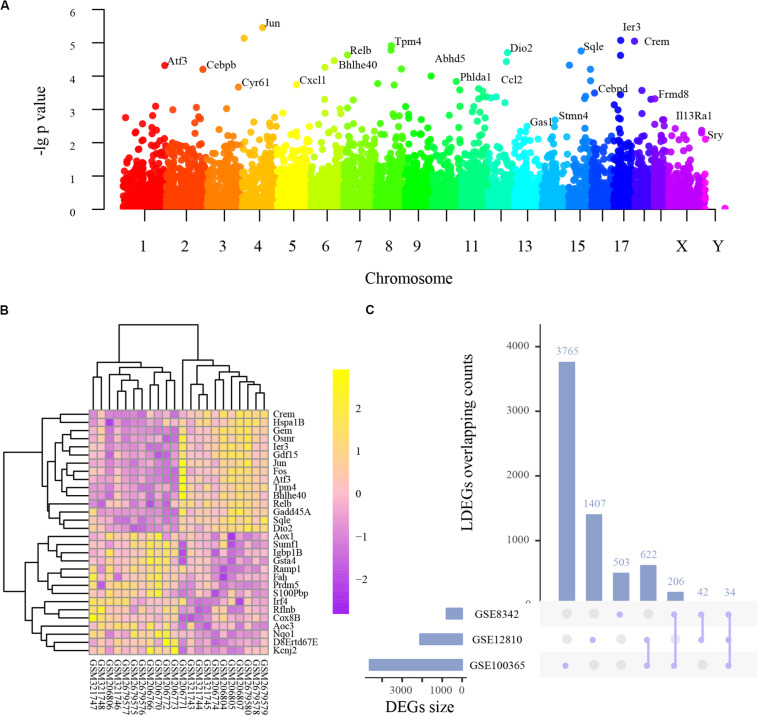
The basic information of all detected genes and representative differentially expressed genes (DEGs). **(A)** Manhattan plot of all genes including the DEGs. The abscissa indicates the chromosomes, and the ordinate represents the p values of those genes. The most significant gene of each chromosome is labeled respectively. **(B)** Heatmap of the top 30 upregulated and downregulated genes. Purple indicates relatively lower expression, and yellow indicates relatively higher expression. **(C)** The overlapping counts of DEGs in the three studies. The abscissa indicates the seven possible sets, and the ordinate suggests to which set the number of DEGs affiliate.

Integrated analysis of the three studies indicated 512 upregulated and 166 downregulated genes, among which 105 were transcription factors (Supplementary Table S1B), and the following PPI network construction and hub genes detection were based on these DEGs. As shown in Figure 1A, the Manhattan plot indicated the parameter of p values and affiliated chromosomes of all genes including the DEGs. The top 15 upregulated genes included cAMP responsive element modulator (Crem), heat shock protein 1B (Hspa1b), GTP binding protein (Gem), oncostatin M receptor (Osmr), immediate early response 3 (Ier3), growth differentiation factor 15 (Gdf15), jun proto-oncogene (Jun), FBJ osteosarcoma oncogene (Fos), activating transcription factor 3 (Atf3), tropomyosin 4 (Tpm4), basic helix-loop-helix family, member e40 (Bhlhe40), reticuloendotheliosis viral oncogene related B (Relb), growth arrest and DNA-damage-inducible 45 alpha (Gadd45a), squalene epoxidase (Sqle), and iodothyronine deiodinase 2 (Dio2). The top 15 downregulated genes included aldehyde oxidase 1 (Aox1), sulfatase modifying factor 1 (Sumf1), immunoglobulin binding protein 1b (Igbp1b), glutathione S-transferase alpha 4 (Gsta4), receptor activity modifying protein 1 (Ramp1), fumarylacetoacetate hydrolase (Fah), PR domain containing 5 (Prdm5), S100P binding protein (S100pbp), interferon regulatory factor 4 (Irf4), refilin B (Rflnb), cytochrome c oxidase subunit 8B (Cox8b), amine oxidase, copper containing 3 (Aoc3), NAD(P)H quinone dehydrogenase 1 (Nqo1), DNA segment Chr 8 ERATO Doi 67 expressed (D8ertd67e), and potassium voltage-gated channel subfamily J member 2 (Kcnj2) (Figure 1B).

These top genes could construct a network that has significantly more interactions than expected (*p* < 0.0001) (Supplementary Figure S1D). The mainly involved biological processes include response to stimulus, cellular process, response to abiotic stimulus, circadian rhythm, and response to external stimulus. The most enriched Kyoto Encyclopedia of Genes and Genomes (KEGG) pathways are Tyrosine metabolism, MAPK signaling pathway, HTLV-I infection, Fluid shear stress and atherosclerosis, and Colorectal cancer (Supplementary Table S1F). These results indicated that noise-induced cochlear pathology might have similar mechanisms with other diseases, such as cancer and atherosclerosis.

### Analysis of Series-Based DEGs

As frequently measured DEGs are more reliable for diagnosing NIHL, we analyzed series-based DEGs. Individual DEG analysis indicated that 34 overlapped in all three studies and 870 in two studies, with overlapping counts shown in Figure 1C.

Based on Youden’s J statistic and the AUC calculation (AUC ranged from 0.84 to 1.00), we ranked these 34 DEGs (Supplementary Table S2). Among the 34 DEGs, 13 displayed significant diagnostic value in the same direction (upregulated or downregulated), including Crem, casein kinase 2 alpha 2 (Csnk2a2), cleavage stimulation factor subunit 3 (Cstf3), cysteine rich angiogenic inducer 61 (Cyr61), Hspa1b, interleukin 13 receptor subunit alpha 1 (Il13ra1), Jun, pleckstrin homology domain containing family H member 1 (Plekhh1), purine rich element binding protein A (Pura), Ras association domain family member 1 (Rassf1), Relb, S100pbp, and tropomodulin 3 (Tmod3) (Table 1). We present the expression levels of these genes in Supplementary Figure S1C, and the statistical results in Table 1.

**TABLE 1:** Thirteen differentially expressed genes displayed significant diagnostic value in the same direction.

**Gene**	**J1**	**J2**	**J3**	**AUC1**	**AUC2**	**AUC3**	**TOP**	**TF**	**HG**
Crem	7.220904	5.386261	6.545	0.88	0.8889	1	Y	Y	Y
Csnk2a2	6.553653	7.273568	3.46	0.96	1	1	N	N	N
Cstf3	7.503475	7.361176	8.55	0.92	1	1	N	N	Y
Cyr61	7.979865	3.887051	7.28	1	1	0.8889	N	N	N
Hspa1b	9.530462	3.392444	6.44	0.92	0.8889	1	Y	N	Y
Il13ra1	6.634921	4.304466	5.07	1	1	1	N	N	N
Jun	8.993056	7.038203	7.25	0.96	1	1	Y	Y	Y
Plekhh1	5.264403	4.338483	6.34	0.84	1	1	N	N	N
Pura	10.53739	5.376438	10.055	0.96	1	1	N	Y	N
Rassf1	7.538773	6.913554	5.84	0.96	1	0.8889	N	N	N
Relb	4.052692	3.586543	5.885	0.96	0.8889	1	Y	Y	Y
S100pbp	8.250666	4.456705	7.555	0.88	1	1	Y	N	N
Tmod3	12.055	3.754847	7.535	0.96	1	1	N	N	N

*J1, J2, J3 mean Youden’s J statistics in three enrolled studies, and AUC1, AUC2, AUC3 mean area under curve, respectively. Y(yes) or N(no) indicates whether respective gene is in TOP (TOP30 DEGs), TF (Transcription Factors), HG (Hub Genes) or not.*

These DEGs could also construct a network that has significantly more interactions than expected (*p* = 0.0228) (Supplementary Figure S1E). The mainly involved biological processes include regulation of cell cycle, circadian rhythm, negative regulation of the cellular process, regulation of cell cycle process, and myeloid cell differentiation. The most enriched KEGG pathways are Epstein-Barr virus infection, MAPK signaling pathway, Mitophagy – animal, HTLV-I infection, and NF-kappa B signaling pathway (Supplementary Table S1G). These results indicated that noise-induced cochlear pathology might be an innate immune response and might have similarities with the infection of Epstein-Barr virus or HTLV-I.

### Pooled Functional Annotation

**FIGURE 2:**
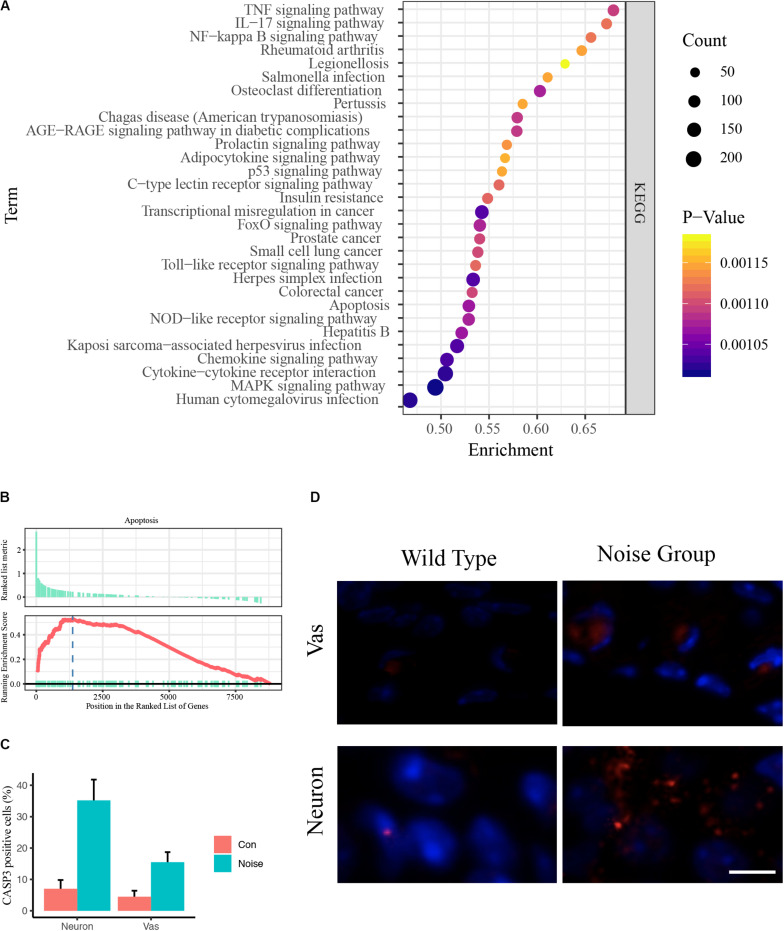
Pooled functional annotation and validation. **(A)** KEGG pathway enrichment analysis of all differentially expressed genes. The abscissa represents the enrichment scores, and the ordinate suggests KEGG terms. **(B)** Gene set enrichment analysis (GSEA) plot of apoptosis. The abscissa represents the ranks of all detected genes, and the ordinate suggests the running score. Most apoptosis molecules lie in the upregulated position, indicating an upregulation of apoptosis in NIHL. **(C)** Quantitative analysis of CASP3 positive cells in wild-type and noise-exposed cochlea. Data are shown as Mean ± SEM. Vas: stria vascularis. **(D)** Representative image of CASP3 IF staining in spiral ganglion area and stria vascularis area of wild-type **(left)** and noise-exposed **(right)** cochlea. Vas: stria vascularis. Scale bar: 10 μm.

To avoid type two error in further analyses, we subjected the DEGs to functional annotation using the clusterProfiler R package (Yu et al., 2012). Enriched KEGG pathways included the TNF signaling pathway, the IL-17 signaling pathway, the NF-kappa B signaling pathway, rheumatoid arthritis, legionellosis, Salmonella infection, osteoclast differentiation, pertussis, Chagas disease, the AGE-RAGE signaling pathway, the prolactin signaling pathway, the adipocytokine signaling pathway, the p53 signaling pathway, the C-type lectin receptor signaling pathway, insulin resistance, transcriptional misregulation in cancer, the FoxO signaling pathway, prostate cancer, small cell lung cancer, the Toll-like receptor signaling pathway, herpes simplex infection, colorectal cancer, apoptosis, the NOD-like receptor signaling pathway, hepatitis B, Kaposi sarcoma-associated herpesvirus infection, the chemokine signaling pathway, cytokine-cytokine receptor interaction, the MAPK signaling pathway, and human cytomegalovirus infection (Figure 2A and Supplementary Table S1C).

We noticed that inflammation-associated pathways occurred frequently, including TNF, IL-17, NF-kappa B, and Toll-like receptor signaling pathways (Cai et al., 2014). These results coincide with previous studies (Tornabene et al., 2006; Moon et al., 2007; Vethanayagam et al., 2016), indicating the enormous impact of inflammation in auditory organs. We also observed that apoptosis was upregulated (Figure 2B). The Caspase 3 IF staining showed that the caspase 3 positive cells were significantly increased in noise group, especially in neurons and stria vascularis areas (Figures 2C,D).

### Gene Co-expression Network and PPI Network

**FIGURE 3:**
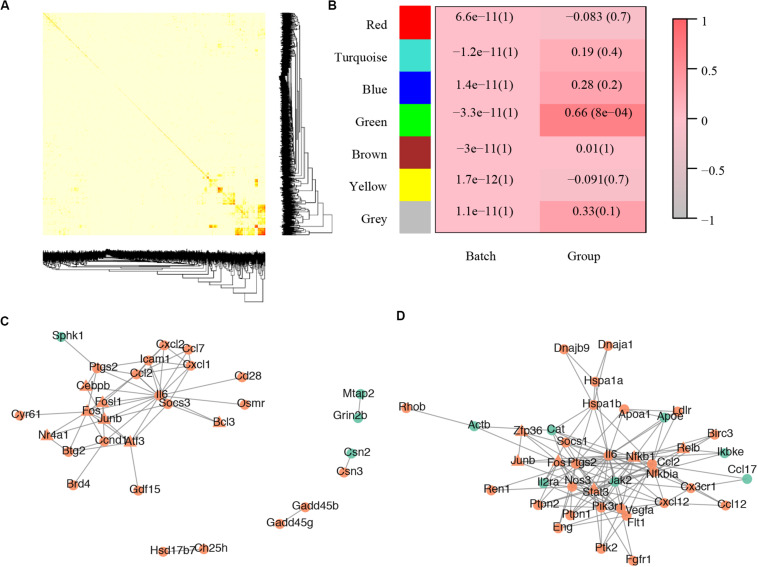
Core modules of noise-induced hearing loss pathogenesis. **(A)** Heatmap plot of network topology. In the right and bottom are gene dendrogram and module assignment. A light color denotes low topological overlap, and progressively darker red denotes higher topological overlap. Darker squares along the diagonal correspond to modules. Seven modules were detected. **(B)** Module-trait correlation. Each cell contains the respective correlation and *p*-value. Red represents high correlation. **(C)** Protein–protein interaction of molecules in the most enriched WGCNA module. **(D)** Protein–protein interaction of the most enriched cluster of all differentially expressed genes.

For the gene co-expression network, a soft threshold of 10 was selected with the correlation coefficient set at 0.85 (Supplementary Figure S2A) and seven modules were detected by the dynamic tree cut (Supplementary Figure S2B). A topological overlap profile of these modules was shown in Figure 3A, darker squares along with the diagonal corresponding to modules. The relationship between the detected modules and mouse group (control or treated) was also calculated and modules with insufficient relationship values were abandoned (Figure 3B). The PPIs of the most enriched WGCNA module (green, Figure 3B) was shown in Figure 3C. A total of 92 nodes and 117 protein pairs were obtained with a combined weight score >0.4.

For the PPI network, a total of 2,945 pairs were detected (Supplementary Table S1E) and improved network visualization of them was presented in Supplementary Figure S3 with a combined score set at more than 0.9. The most enriched PPI module (score = 13.45, nodes = 41, edge = 269) indicated 41 susceptible molecules (Figure 3D) including Relb, Hspa1b, C-C motif chemokine ligand 2 (Ccl2), prostaglandin-endoperoxide synthase 2 (Ptgs2), low density lipoprotein receptor (Ldlr), plasminogen activator tissue type (Plat), and chemokine C-C motif ligand 17 (Ccl17). These DEGs could also construct a network that has significantly more interactions than expected (*p* < 0.0001) (Supplementary Figure S1F).

### Functional Annotation of WGCNA and PPI Enriched Modules

**FIGURE 4:**
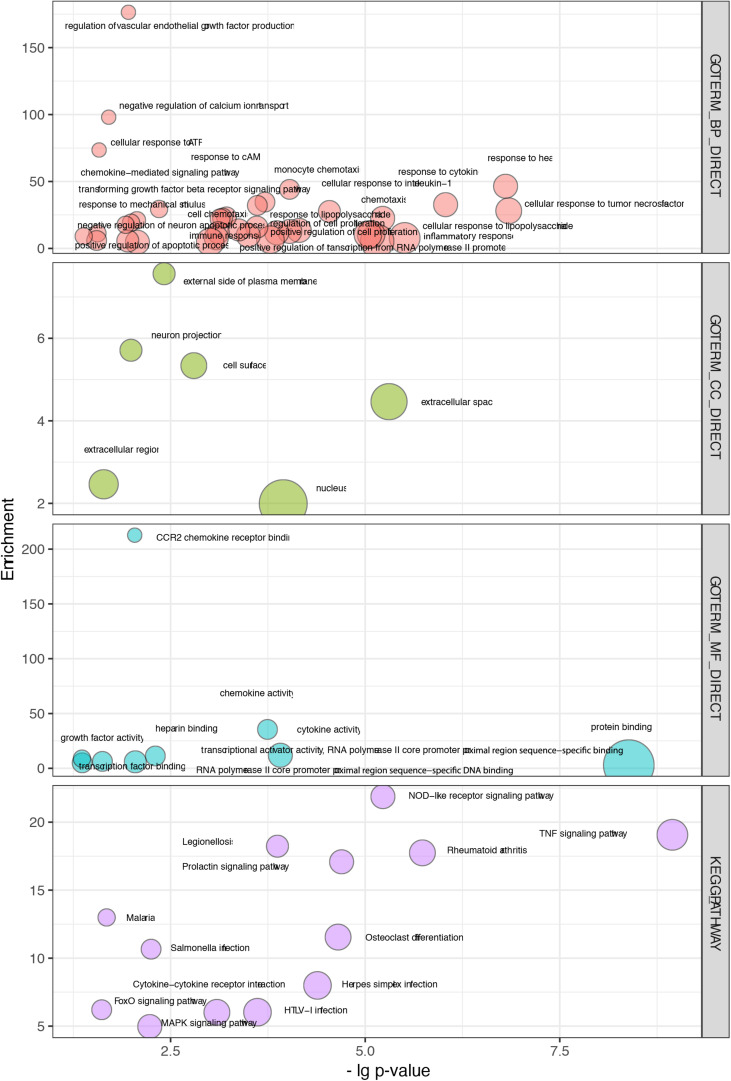
Overlapping terms of GO and KEGG pathway enrichment in WGCNA and PPI modules. The abscissa indicates the *p* values, and the ordinate represents the enrichment scores of those terms.

To explore the biological relevance of these modules, the genes were subjected to Gene Ontology (GO) functional and KEGG pathway enrichment analyses. Thirteen overlapping pathways between the two modules included the TNF signaling pathway, rheumatoid arthritis, the NOD-like receptor signaling pathway, the prolactin signaling pathway, Salmonella infection, cytokine-cytokine receptor interaction, the MAPK signaling pathway, HTLV-I infection, malaria, herpes simplex infection, osteoclast differentiation, legionellosis, and the FoxO signaling pathway (Figure 4 and Supplementary Table S1D).

Thirty-seven biological processes were consistently observed, indicating several potential responses, including the response to cytokines, response to heat, response to cAMP, response to ATP, immune response, and the response to mechanical stimulus (Figure 4 and Supplementary Table S1D). Six cellular components were consistently observed, suggesting that the main molecular and biological changes occur in the external side of the plasma membrane, extracellular region, and nucleus (Figure 4 and Supplementary Table S1D). Nine overlapping molecular function terms indicated that binding activities might be important. Those activities included CCR2 receptor binding, protein binding, and transcription factor binding (Figure 4 and Supplementary Table S1D).

Interestingly, our data were consistent with pooled annotations (Supplementary Table S1C). Notably, IL6, NFKBIA, NFKB1, NOS3, PTPN1, PIK3R1, and STAT3 (members in the enriched WGCNA module) have been shown to alter insulin resistance (Seo et al., 2016). Researchers have shown that insulin resistance can decrease the number of ribbon synapses and elevate the ABR threshold in an age-related hearing loss model without affecting OHCs, IHCs, and SGNs (Yu et al., 2015).

### Experimental Validation of Susceptible Hub Genes

**FIGURE 5:**
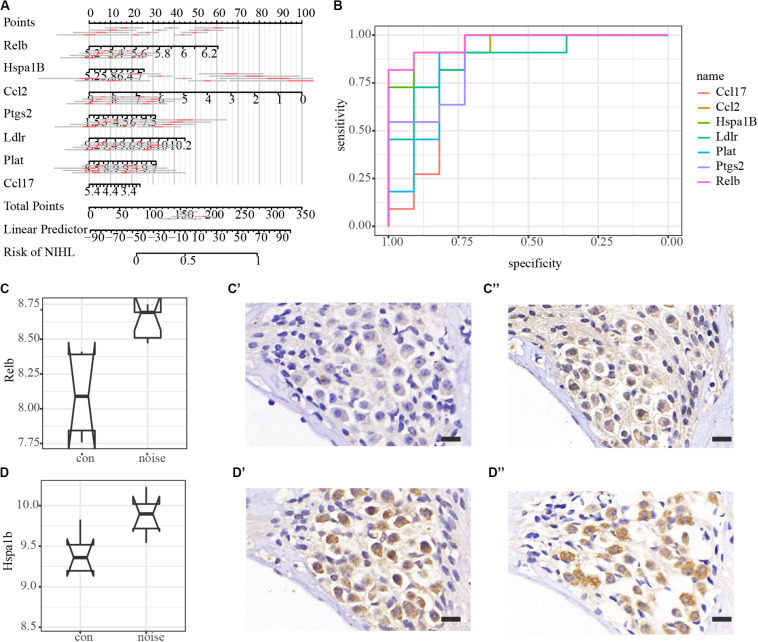
Logistic regression, diagnostic value, and immunohistochemistry validation of hub genes. **(A)** Nomogram plot of the most susceptible molecules (*p* < 0.001) to visualize logistic regression in the PPI module. **(B)** Receiver operating characteristic (ROC) curves of hub genes. AUCs of Relb, Hspa1b, Ccl2, Ptgs2, Ldlr, Plat, and Ccl17 are 0.9669, 0.9587, 0.8926, 0.8843, 0.876, 0.8678, and 0.8347, respectively. **(C–C”)** Immunohistochemical validation of Relb in the spiral ganglion area. Scale bar: 15 μm. **(D–D”)** Immunohistochemical validation of Hspa1b in the spiral ganglion area. Scale bar: 15 μm.

All PPI susceptible molecules were utilized in logistic regression (stepwise) to predict NIHL as shown in Figure 5A. Logistic regression predicted that Ln OR = −19.078 + Relb × 1.608 + Hspa1b × 0.308 + Ccl2 ×−0.227 + Ptgs2 × 0.155 + Ldlr × 0.814 + Plat × 0.813 + Ccl17 ×−0.364 (Multiple R-squared: 0.9822). The AUCs for Relb, Hspa1b, Ccl2, Ptgs2, Ldlr, Plat, and Ccl17 were 0.9669, 0.9587, 0.8926, 0.8843, 0.876, 0.8678, and 0.8347, respectively, with the receiver operating characteristic curve presented in Figure 5B. We hypothesized that Relb, Hspa1b, Ccl2, Ptgs2, Ldlr, Plat, and Ccl17 were hub genes with diagnostic value at the transcription level, and Relb and Hspa1b IHC were assessed to validate our hypothesis (Figures 5C,D) at the translation level. While Hspa1b may have post-transcription modifications, the IHC results of Relb were quantitatively consistent with the microarray results.

## Discussion

Prevention and treatment are two strategies used against NIHL. However, prevention strategies, including hearing conservation programs, education, and ear protection devices are ineffective because of their lack of efficiency and accessibility (Chen and Tsai, 2003; Rajguru, 2013; Sekhar et al., 2014).

High doses of corticoids (a widely used anti-inflammatory hormone) are often prescribed by physicians after an acoustic trauma to mitigate the inflammatory response. In 2006, researchers revealed that stressors (sound, heat, or stress), as a pre-emptive medication, could increase the receptiveness of glucocorticoids by increasing the number of receptors of the anti-inflammatory hormone (Tahera et al., 2007). It reduces the inflammation caused by the acoustic trauma, which causes subsequent damage to hair cells.

For NIHL treatment, a series of clinical trials have been conducted. Intratympanical injection with AM-111 may have a therapeutic effect in cases of acute trauma after firecracker exposure (Suckfuell et al., 2007). Immediate treatment with a combination of prednisolone and piracetam appeared to rescue patients with acute trauma after exposure to gunshots, as significantly lower threshold shifts were observed (Psillas et al., 2008). In recent clinical trials, antioxidants that reduce reactive oxygen after traumatic noise events appear promising (Prasher, 1998). Ebselen has been shown to have promising results for both TTS and PTS by reducing the threshold shift (Lynch and Kil, 2005; Satheeshkumar and Mugesh, 2011; Kil et al., 2017).

Research has indicated that RelB, a member of the NF-kappaB family, is upregulated at the transcription level and results in increases in the lateral wall and the rest area after acoustic overstimulation (Yamamoto et al., 2009). Notably, the expression of adhesion molecules (Icam1, Icam5, and Nrcam) and iNOS was observed in tissues around the capillaries in the stria vascularis, indicating hemodynamics and changes in the cellular integrity (Supplementary Table S1B).

After acoustic overstimulation, heat shock protein (HSP) induction was observed in the cochlea (Lim et al., 1993). Unsurprisingly, HSP induction protected against noise trauma in guinea pig cochlea (Mikuriya et al., 2005). In contrast, mice deficient in heat shock factor 1 (the major transcription factor triggering HSP expression) exhibited a decreased TTS recovery ability following noise overstimulation (Fairfield et al., 2005). Notably, HSP70-2 (Hspa1B) polymorphism (rs1061581) was the only HSP70 polymorphism that had a significant association with NIHL in both Chinese and Swedish sample sets (Yang et al., 2006; Konings et al., 2009), which is thought to be evidence for NIHL susceptibility.

The mitochondrial Ptgs2 (COX2) mutation is associated with aminoglycoside antibiotic-induced deafness in a Han Chinese pedigree (Chen et al., 2013). Inhibition of cyclooxygenase-2 (COX2) by NS398 attenuates NIHL in mice (Sun et al., 2016), whereas celecoxib does not (Pourbakht, 2013; Li et al., 2015).

Ccl family members (such as Ccl2 and Ccl17)-associated inflammatory responses may be detrimental to hearing recovery, suggesting their role as potential post-insult therapeutic targets for treatment (Fujioka et al., 2014).

Currently, no clinical treatments exist to reverse the effects of permanent NIHL (Oishi and Schacht, 2011). Limitations of our present study include the following: first, validations for the detected molecules and pathways were still needed, and technologies with higher resolution such as single cell RNA-seq and spatial transcriptome would facilitate our study; second, even if the pathways influence NIHL pathogenesis, it is unclear how to make treatments safer, more convenient, and accessible. However, given that molecular imaging is utilized as a powerful tool in the investigation of parkinsonian disorders, the identification of biomarkers of early changes remains a challenge to predict the clinical trajectory of these disorders (Strafella et al., 2017). Exciting new tracer developments are aiding the investigation of *in vivo* markers in its treatment (Géléoc and Holt, 2014). Similarly, NIHL and even all sensorineural hearing loss will benefit from these technologies. The first step is to identify potential markers, and our current research does so in a comprehensive and systematic way.

In conclusion, our results suggest that genes involved in multiple inflammatory pathways are influenced most significantly after noise exposure. Relb, Hspa1b, Ccl2, Ptgs2, Ldlr, Plat, and Ccl17 are crucial for NIHL pathogenesis in mouse models. While further validation studies are required, these genes could potentially be used as novel diagnostic and therapeutic targets for NIHL.

## Data Availability Statement

The datasets presented in this study can be found in online repositories. The names of the repository/repositories and accession number(s) can be found in the article/ Supplementary Material.

## Ethics Statement

The animal study was reviewed and approved by XinHua Hospital Affiliated to Shanghai Jiao Tong University School of Medicine.

## Author Contributions

MX and QW contributed for initiation and conduction. QW contributed for the writing of the manuscript. QW, YS, BY, HH, and CF performed the experiments. BY, QW, and YS processed and analyzed the data. All authors contributed to the article and approved the submitted version.

## Conflict of Interest

The authors declare that the research was conducted in the absence of any commercial or financial relationships that could be construed as a potential conflict of interest.

## Footnotes

1 https://www.ncbi.nlm.nih.gov/geo

2 https://www.ebi.ac.uk/arrayexpress

3 https://string-db.org/

## Abbreviations

AE: ArrayExpress
AUC: area under curve
DEGs: differentially expressed genes
GEO: Gene Expression Omnibus
GO: gene ontology
KEGG: Kyoto encyclopedia of genes and genomes
NIHL: noise-induced hearing loss
OR: odds ratio
PTS: permanent threshold shift
ROS/RNS: reactive oxygen/nitrogen species
TTS: temporary threshold shift
TOM: topological overlap matrix
WGCNA: weighted gene co-expression network analysis.
