# Supplementary figures and images for: Systematic Transcriptome Analysis of Noise-Induced Hearing Loss Pathogenesis Suggests Inflammatory Activities and Multiple Susceptible Molecules and Pathways
Source: Front Genet. 2020 Aug 28;11:968. doi: 10.3389/fgene.2020.00968 (PMC7483666; doi:10.3389/fgene.2020.00968)

A

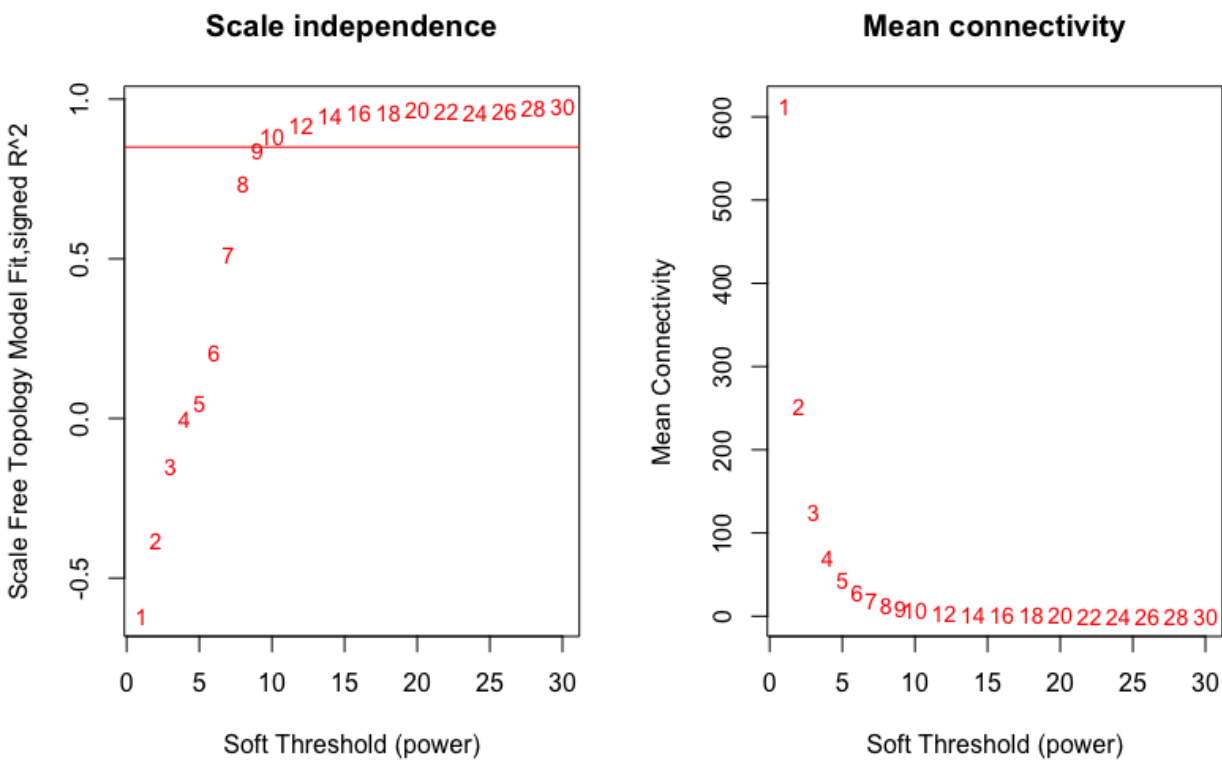

B

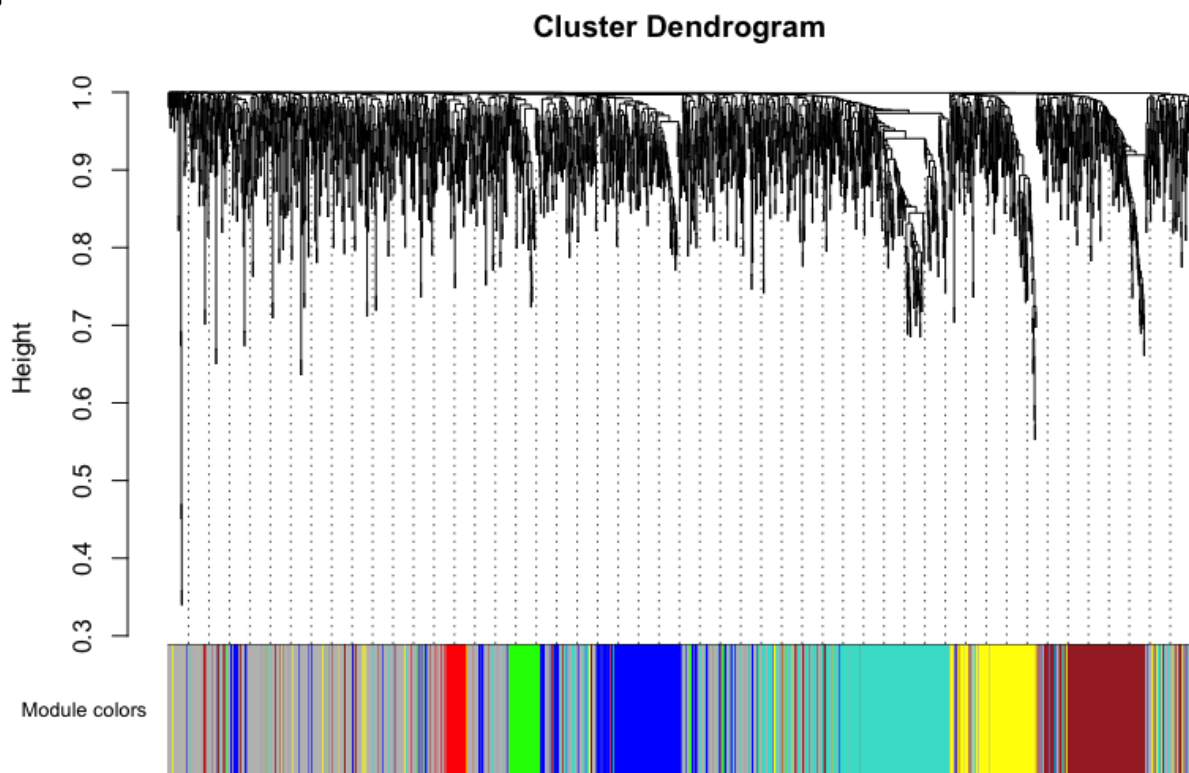

Supplement: Supplementary file 4 [file Data_Sheet_2.PDF]

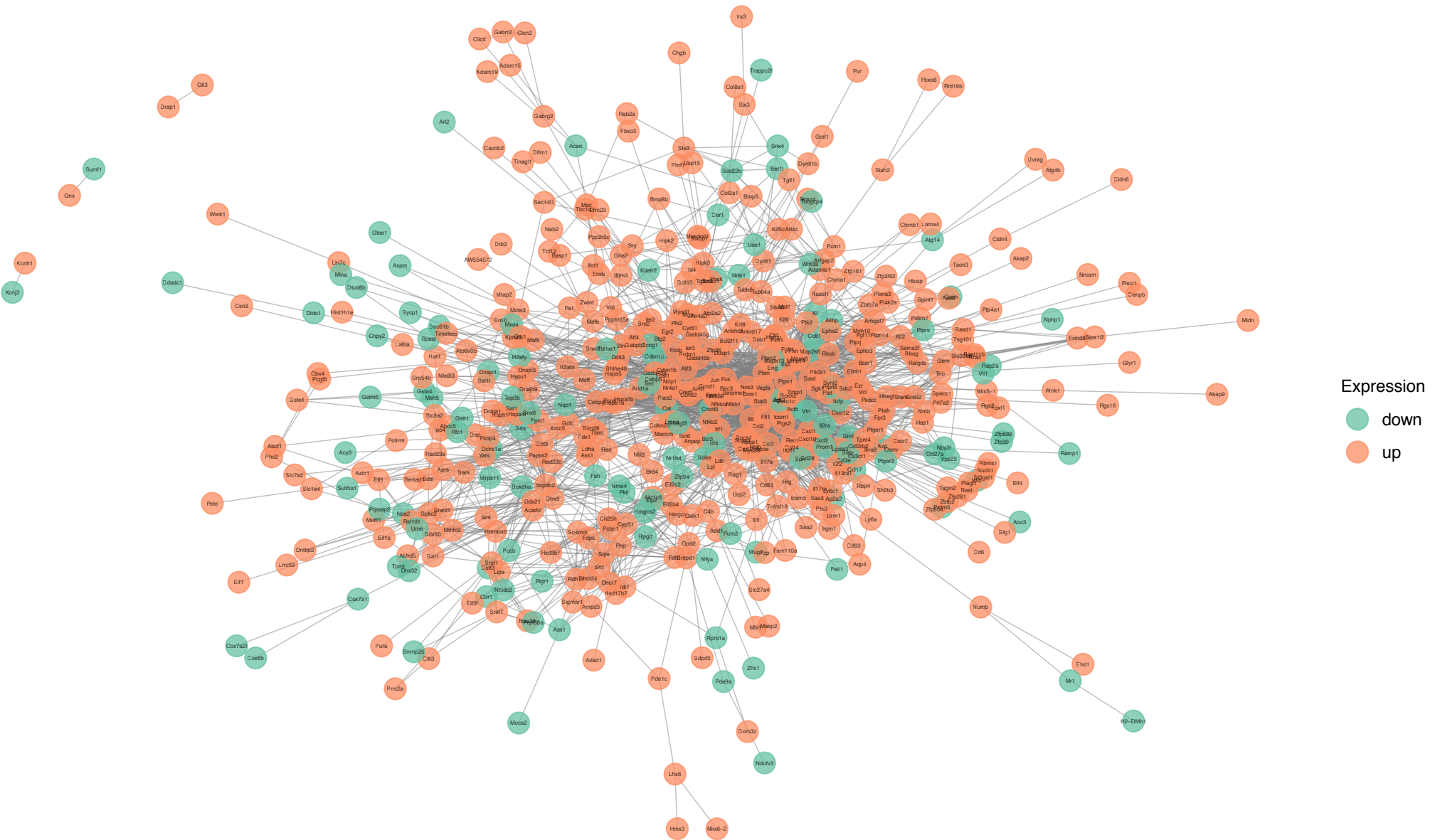

Supplement: Supplementary file 5 [file Data_Sheet_3.PDF]
