# Supplementary material for: Systematic Transcriptome Analysis of Noise-Induced Hearing Loss Pathogenesis Suggests Inflammatory Activities and Multiple Susceptible Molecules and Pathways
Source: Front Genet. 2020 Aug 28;11:968. doi: 10.3389/fgene.2020.00968 (PMC7483666; doi:10.3389/fgene.2020.00968)
Supplement: Supplementary file 3 [file Data_Sheet_1.PDF]

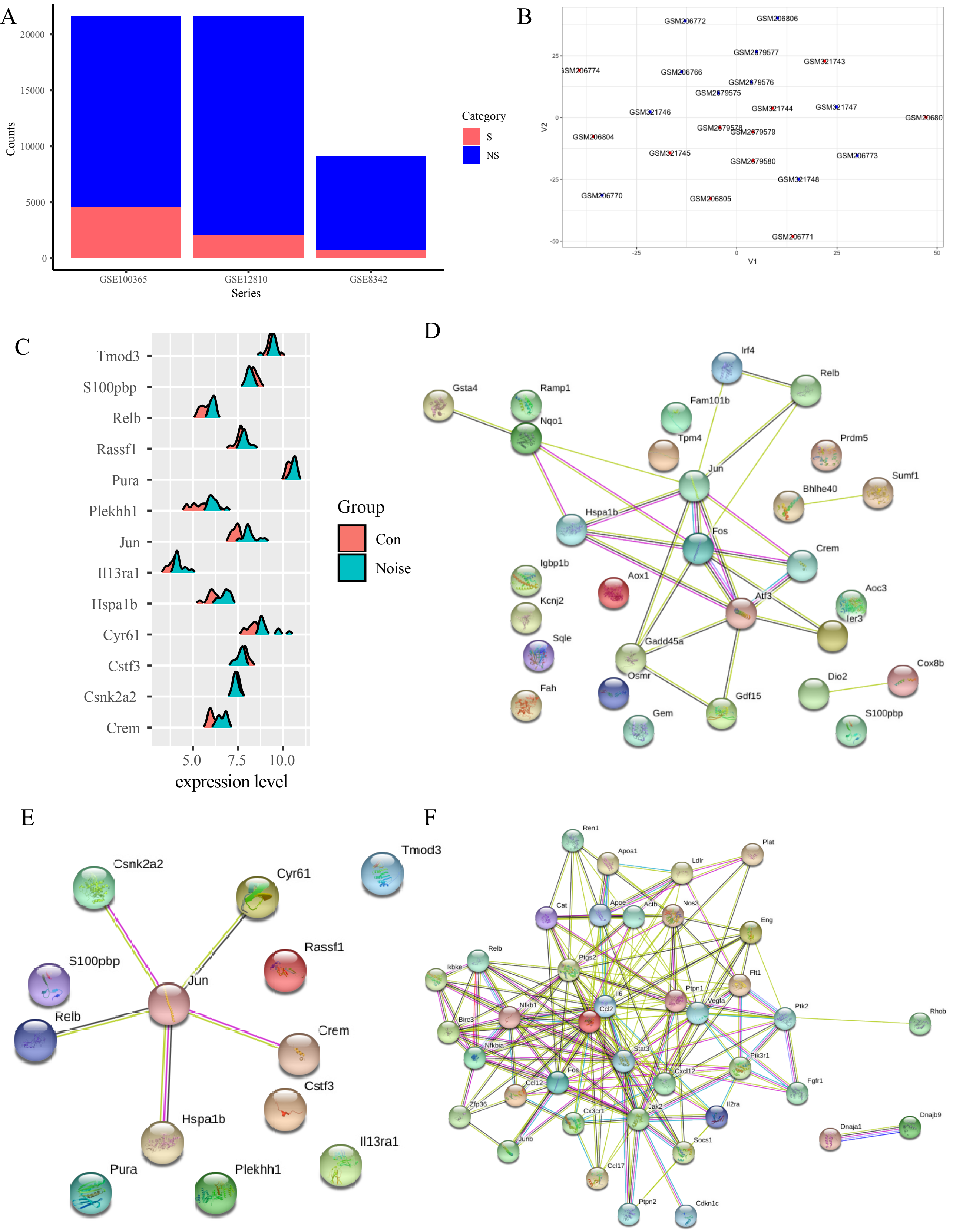
A
Counts
20000
15000
10000
5000
0
GSE100365
GSE12810
GSE8342
Series
Category
S
NS
B
V2
V1
25
0
-25
-50
50
GSM206772
GSM206806
GSM2679577
GSM321743
GSM206774
GSM206766
GSM2679576
GSM2679575
GSM321746
GSM321744
GSM321747
GSM20680
GSM206804
GSM2679578
GSM2679579
GSM321745
GSM2679580
GSM206773
GSM321748
GSM206770
GSM206805
GSM206771
C
Tmod3
S100pbp
Relb
Rassf1
Pura
Plekhh1
Jun
Il13ra1
Hspa1b
Cyr61
Cstf3
Csnk2a2
Crem
5.0
7.5
10.0
expression level
Group
Con
Noise
D
Irf4
Relb
Gsta4
Ramp1
Fam101b
Nqo1
Tpm4
Prdm5
Jun
Sumf1
Bhlhe40
Hspa1b
Fos
Crem
Igbp1b
Aox1
Atf3
Aoc3
Kcnj2
Ier3
Sqle
Gadd45a
Osmr
Cox8b
Dio2
Fah
Gdf15
Gem
S100pbp
E
Tmod3
Csnk2a2
Cyr61
Rassf1
S100pbp
Jun
Relb
Crem
Cstf3
Hspa1b
Il13ra1
Pura
Plekhh1
F
Ren1
Plat
Apoa1
Ldlr
Cat
Apoe
Actb
Nos3
Eng
Relb
Ptgs2
Ikbke
Flt1
Il6
Ptpn1
Nfkb1
Ccl2
Vegfa
Ptk2
Rhob
Birc3
Nfkbia
Stat3
Pik3r1
Fos
Cxcl12
Fgfr1
Ccl12
Zfp36
Cx3cr1
Jak2
Il2ra
Dnajb9
Dnaja1
Junb
Socs1
Ccl17
Ptpn2
Cdkn1c
